# Assessment of macro, trace and toxic element intake from rice: differences between cultivars, pigmented and non-pigmented rice

**DOI:** 10.1038/s41598-024-58411-1

**Published:** 2024-05-06

**Authors:** Xingyong Liu, Qian Li, Benlin Yin, Hongmei Yan, Yunmei Wang

**Affiliations:** Institute of Quality Standards and Testing Technology, Yunnan Academy of Agricultural Sciences, Kunming, 650000 China

**Keywords:** Rice, Essential micronutrients, Toxic elements, Pigmented rice, Health risk, Environmental sciences, Health care, Risk factors

## Abstract

Pigmented and non-pigmented rice varieties (grown in different areas) were collected in China, Yunnan, to investigate the content of macro-, trace elements and potentially toxic elements (PTEs), and to assess the health risk associated with dietary intake. The order of elemental concentrations in rice was Mn > Zn > Fe > Cu > Se for trace elements, P > K > Mg > Ca > Na for macro elements, and Cr > As > Cd for PTEs. Rice with a high concentration of essential elements also associated with a high content of PTEs. In addition, higher content of Cr, Mn and Na were found in pigmented rice. The health risk assessment showed that the daily intake of all elements was below the tolerable limit (UL). Moreover the intake of Fe, Zn and Se was far from sufficient for the nutrient requirement. The PTEs in rice dominated the health risk. Of concern is that this rice consumption is likely to contribute to carcinogenic risks in the long term and that adults are at higher health risk from pigmented rice compared to non-pigmented rice. This study confirms that the lack of essential micronutrients in rice and the health risk associated with rice diets should remain a concern.

## Introduction

Rice is the staple food for more than half of the world's population, meeting their daily nutritional needs. In recent decades, due to environmental pollution, there has been an accumulation of various potentially toxic elements (PTEs) in the rice plant^1^, and contaminated rice is the major source of human exposure to PTEs^2^. On the other hand, deficiency of the essential elements Fe and Zn in rice is most common worldwide, and populations that consume mainly rice can easily become micronutrient deficient^3^. The accumulation of PTEs and micronutrient deficiencies in rice grain is an enduring issue.

Consumption of rice contaminated with PTEs disrupts normal physiological/molecular mechanisms and causes various health problems. One study showed that the dietary intake of Cd from rice in the Chinese population ranged from 430 to 930 μg kg^−1^ BW day^−1^^4^. Prolonged exposure to high Cd and Cr concentrations can lead to respiratory damage, kidney function, impaired immune function, bone loss, endocrine disruption, etc^5^. High As concentrations can lead to intestinal microbiome disorders and liver cancer^6^ and Hg can lead to nervous system damage in adults and impaired neurological development in infants and children^7^. Pb impairs the nervous system and leads to mental retardation^8^. In addition, micronutrient deficiencies cause "hidden hunger", such as Fe deficiency causes anaemia^9^, Zn leads to stunting, poor quality of pregnancy and birth weight^10^. Deficiency of Se leads to Keshan disease, poor immune function and fertility, insufficient supply of Mn leads to growth disorders, ataxia and low reproductive function^11^.

In following the trends of safety and nutrition, the daily consumption of rice must meet the nutritional requirements necessary for the human body, but also prevent and control the health effects of harmful elements. The level of heavy metal contamination in rice is diverse and varies regionally in composition due to natural background levels in soil, industrialization, and other anthropogenic activities. Rice grains have a high ability to accumulate heavy metals, and their levels in rice depend on growing conditions. Huang^12^ reported that Cd and As were the major non-carcinogenic and carcinogenic factors in rice from Hubei, China. Li^13^ reported that Cd, Cu, Zn, and Ni had a different spatial contamination pattern in rice, and that Pb and Cd were the main health risk^14^. In addition, higher than the acceptable level (1 × 10^−6^) for the carcinogenic risk of As was found in rice samples from Iran^15^, but Takamoto A^16^ found that Cd and As in milled rice grown in Japan, Vietnam, and Indonesia do not pose a non-carcinogenic health risk, and same result by Bielecka^17^. In addition, the interactions between heavy metal elements may also affect the migration and accumulation of heavy metals in soil or paddy rice^18^. For example, the accumulation of Si in rice plants likely reduces the translocation of Cu and Cd from soil to grain^19^, and iron oxide deposition in the rhizosphere may serve as a barrier that prevents Pb in soil at the root surface and leads to lower Pb accumulation in brown rice^20^.

Rice produced in Yunnan is mainly used for processed products. Therefore, comprehensive and systematic studies are needed to evaluate the health risks of the content of PTEs and essential elements in rice. Shi^21^ reported that vegetables in Yunnan have relatively high exceedances of Pb and Cd. However, little information is known about PTEs from rice in Yunnan. Furthermore, previous studies only focused on the health risks of PTEs in rice and rarely focused on the essential elements.

In order to implement effective strategies to improve rice quality, it is of great importance to know more about the basic information on the situation and health risks of heavy metals and macro and trace elements of local rice at the place of origin, variety and type. Consequently, this study aims to: (1) analyze the rice concentration of PTEs, macro minerals (Na, P, K, Mg and Ca) and trace elements (Mn, Zn, Fe, Cu and Se) from Yunnan, China; (2) rice element differences between rice production area, pigmented and non-pigmented; (3) evaluate the potential health risks of high quality rice commonly used in the local market. The results would be valuable for the adaptation of suitable growing areas and varieties, useful for the control of heavy metals and the enrichment of trace elements.

## Results

The analytical data for elements in the 20 rice varieties from different areas have been summarised as Tables 1,2. The concentrations of macro and trace elements in the samples studied occurred respectively in the following orders: P > K > Mg > Ca > Na and Mn > Zn > Fe > Cu > Se (Table 1), and the order of concentrations of toxic metals was Cr > As > Cd (Table 2).

**Table 1:** Contents of macro and trace element in rice samples.

Sample number	Rice varieties	Ca mg/kg	Cu mg/kg	Fe mg/kg	K mg/kg	Mg mg/kg	Mn mg/kg	Na mg/kg	P mg/kg	Zn mg/kg	Se μg/kg
1	bianhua red soft rice	114.80 ± 1.97^a^	0.67 ± 0.03^f^	11.49 ± 0.58^a^	2564.00 ± 65.05^a^	1239.42 ± 20.95^a^	17.53 ± 0.55^c^	8.45 ± 0.09^e^	3341.38 ± 67.78^a^	17.05 ± 0.38^b^	64.00 ± 83.14^a^
2	Yuanyang red rice	87.54 ± 1.14^c^	1.08 ± 0.06^d^	7.25 ± 0.10^e^	1219.13 ± 59.25^g^	555.08 ± 17.30^f^	14.41 ± 0.84^d^	10.46 ± 0.58^c^	1990.23 ± 29.09^e^	15.61 ± 0.85^cd^	13.67 ± 1.53^d^
3	red glutinous rice1	70.53 ± 2.22^f^	2.50 ± 0.14^a^	3.53 ± 0.08^i^	1432.67 ± 65.55^f^	442.15 ± 10.32^h^	7.15 ± 0.09^f^	7.53 ± 0.12^e^	1420.71 ± 19.58^h^	18.82 ± 1.01^a^	13.33 ± 2.31^bc^
4	Lvchun red rice	101.29 ± 1.86^b^	ND	11.22 ± 0.34^a^	1645.21 ± 50.86^d^	986.49 ± 11.53^d^	12.64 ± 0.98^d^	9.51 ± 0.16^cd^	3034.90 ± 49.06^c^	15.22 ± 0.53^d^	41.67 ± 3.79^ab^
5	Red glutinous rice 2	113.02 ± 2.00^a^	ND	7.96 ± 0.17^d^	1530.33 ± 62.52^e^	947.22 ± 12.20^e^	11.87 ± 1.16^de^	7.74 ± 0.10^e^	2799.31 ± 31.30^d^	12.39 ± 0.70^ef^	55.33 ± 6.43^a^
6	Banna purple rice	51.62 ± 0.91^h^	ND	2.41 ± 0.06^k^	942.26 ± 17.49^h^	326.82 ± 15.12^i^	8.80 ± 0.14^f^	6.06 ± 0.10^h^	1071.57 ± 100.65^j^	12.62 ± 0.60^ef^	19.67 ± 2.08^bc^
7	Dehong purple rice	73.58 ± 1.11^e^	1.26 ± 0.09^c^	3.24 ± 0.09^j^	1234.01 ± 26.95^g^	475.03 ± 6.70^g^	6.54 ± 0.25^f^	6.07 ± 0.06^h^	1721.50 ± 77.75f.	16.98 ± 0.45^b^	21.00 ± 2.65^bc^
8	Mojiang purple rice	101.96 ± 3.66^b^	1.11 ± 0.09^d^	8.53 ± 0.13^c^	2095.55 ± 37.20^b^	1111.78 ± 11.43^c^	20.47 ± 0.85^b^	6.64 ± 0.11^fg^	3115.08 ± 50.21^c^	16.57 ± 0.30^bc^	81.33 ± 4.16^a^
9	black rice	39.77 ± 1.54^j^	1.03 ± 0.05^d^	3.84 ± 0.07^h^	1244.27 ± 70.79^g^	422.49 ± 16.93^h^	6.02 ± 0.04^fg^	6.09 ± 0.15^h^	1531.41 ± 98.08^g^	12.63 ± 0.60^ef^	40.33 ± 1.53^ab^
10	purple glutinous rice	111.87 ± 3.43^a^	0.68 ± 0.01f.	9.33 ± 0.08^b^	1825.07 ± 50.87^c^	1147.98 ± 15.13^b^	16.95 ± 0.36^c^	8.12 ± 0.14^e^	3231.69 ± 84.41^b^	17.62 ± 0.48^b^	18.67 ± 4.04^bc^
11	Zhefang rice	45.51 ± 2.73^i^	0.92 ± 0.01^e^	3.10 ± 0.05^j^	537.45 ± 22.07^jk^	166.44 ± 13.26^m^	8.76 ± 0.15^f^	7.15 ± 0.10^ef^	862.97 ± 22.00^k^	15.67 ± 0.61^cd^	62.34 ± 4.93^a^
12	Menghai fragrant rice	38.45 ± 0.79^jk^	0.36 ± 0.01^h^	6.17 ± 0.08f.	565.22 ± 24.02^j^	206.35 ± 9.01^l^	8.64 ± 0.11^f^	31.97 ± 2.72^a^	981.82 ± 17.04^j^	14.12 ± 0.67^e^	20.32 ± 4.16^bc^
13	Lvchun white rice	46.30 ± 1.78^i^	ND	2.18 ± 0.04^k^	627.06 ± 10.67^j^	217.82 ± 13.41^l^	5.11 ± 0.12^gh^	7.23 ± 0.15^ef^	1033.55 ± 63.03^j^	9.61 ± 0.45^g^	26.33 ± 4.04^d^
14	Yunjing 37	57.49 ± 1.71^g^	0.47 ± 0.01^g^	1.07 ± 0.08^m^	633.06 ± 15.34^j^	244.41 ± 9.71^jk^	6.83 ± 0.13^f^	11.46 ± 0.16^c^	861.01 ± 33.96^k^	10.09 ± 0.20^g^	12.35 ± 3.21^d^
15	Yunda 107	67.53 ± 0.81^f^	ND	0.56 ± 0.01^n^	423.87 ± 13.43^lm^	110.39 ± 6.20^n^	4.45 ± 0.07^h^	7.17 ± 0.17^ef^	697.45 ± 14.87^l^	7.64 ± 0.36^i^	4.33 ± 0.58^d^
16	Yunhui 290	42.23 ± 1.20^j^	0.93 ± 0.01^e^	1.71 ± 0.07^l^	802.54 ± 15.71^i^	274.57 ± 7.54^j^	6.20 ± 0.13^fg^	8.05 ± 0.19^e^	1227.09 ± 124.88^i^	14.23 ± 0.77^e^	23.00 ± 3.51^bc^
17	Chujing 39	76.62 ± 0.78^d^	0.93 ± 0.06^e^	4.55 ± 0.08^g^	846.20 ± 10.44^i^	319.47 ± 20.82^i^	105.70 ± 5.31^a^	8.29 ± 0.12^e^	1047.58 ± 54.04^j^	12.17 ± 1.02^f^	20.33 ± 3.51^bc^
18	Chujing 40	52.49 ± 0.99^h^	ND	2.48 ± 0.16^k^	544.87 ± 16.86^jk^	253.87 ± 6.89^j^	5.48 ± 0.14^gh^	11.05 ± 0.34^c^	997.31 ± 19.77^j^	8.63 ± 0.26^gh^	12.00 ± 2.65^d^
19	soft rice 88	46.73 ± 0.98^i^	1.51 ± 0.08^b^	4.11 ± 0.13^h^	410.80 ± 13.75^mn^	218.47 ± 16.77^l^	7.96 ± 0.10^f^	7.74 ± 0.10^e^	943.16 ± 26.61^jk^	13.32 ± 0.19^e^	29.33 ± 3.51^ab^
20	Kunming local white rice	53.12 ± 1.40^h^	ND	1.19 ± 0.05^m^	485.96 ± 11.13^kl^	165.76 ± 10.26^m^	4.76 ± 0.09^gh^	16.74 ± 0.17^b^	796.01 ± 34.06^kl^	8.75 ± 0.25^gh^	6.10 ± 0.36^d^
	Average level	69.62 ± 26.19	1.03 ± 0.53	4.80 ± 3.34	1080.46 ± 603.31	491.60 ± 21.66	14.31 ± 21.66	9.68 ± 5.73	1635.29 ± 915.56	13.48 ± 3.26	29.27 ± 26.14

Results are presented in dry weight as means ± SDs (n = 3). Means within a column with different superscript have differ significantly (*p* < 0.05).

Detection limits: Ca, Fe, K, Mg, Na, P were each at 1 mg kg^−1^; Cu was at 0.05 mg kg^−1^, Mn at 0.1 mg kg^−1^, Zn at 0.5 mg kg^-1^ and Se at 0.01 mg kg^−1^.

*ND* not detectable.

**Table 2:** Contents of toxic elements in rice samples (μg/kg).

Sample number	Rice varieties	Cr	As	Cd
1	Bianhua red soft rice	293.33 ± 20.82^b^	180.00 ± 20.00^ab^	53.67 ± 2.52^e^
2	Yuanyang red rice	170.00 ± 20.00^e^	34.00 ± 5.29^e^	ND
3	Red glutinous rice1	220.00 ± 20.00^c^	123.33 ± 25.17^bc^	12.33 ± 2.51^j^
4	Lvchun red rice	180.00 ± 30.00^cd^	166.67 ± 15.28^ab^	3.33 ± 0.58^j^
5	Red glutinous rice 2	240.00 ± 20.00^c^	240.00 ± 10.00^a^	7.97 ± 0.35^j^
6	Banna purple rice	246.67 ± 30.55^c^	163.33 ± 15.24^ab^	16.31 ± 1.53^fj^
7	Dehong purple rice	246.67 ± 40.41^c^	75.00 ± 5.00^e^	250.00 ± 10.00^a^
8	Mojiang purple rice	230.00 ± 20.00^c^	186.67 ± 20.72^ab^	79.33 ± 4.04^d^
9	Black rice	183.33 ± 15.28^cd^	240.00 ± 20.00^a^	29.33 ± 2.08f.
10	Purple glutinous rice	223.33 ± 30.55^c^	126.67 ± 20.82^bc^	206.67 ± 15.28^b^
11	Zhefang rice	206.67 ± 20.82^c^	196.67 ± 81.45^a^	71.65 ± 4.73^d^
12	Menghai fragrant rice	520.00 ± 45.83^a^	116.67 ± 15.28^d^	3.50 ± 0.50^j^
13	Lvchun white rice	176.67 ± 15.28^d^	126.00 ± 26.23^bc^	1.93 ± 0.12^j^
14	Yunjing 37	243.33 ± 37.86^c^	47.67 ± 5.51^e^	5.00 ± 0.30^j^
15	Yunda 107	223.33 ± 20.82^c^	62.00 ± 3.46^e^	2.97 ± 0.06^j^
16	Yunhui 290	216.67 ± 25.17^c^	53.67 ± 5.51^e^	143.32 ± 35.12^c^
17	Chujing 39	323.33 ± 15.28^b^	63.65 ± 45.71^e^	12.00 ± 2.01^j^
18	Chujing 40	220.00 ± 26.46^c^	60.62 ± 3.51^e^	2.03 ± 0.15^j^
19	Soft rice 88	203.33 ± 15.28^c^	35.00 ± 6.56^e^	47.04 ± 4.58^e^
20	Kunming local white rice	170.00 ± 20.00^e^	54.33 ± 5.51^e^	ND
Average level		236.83 ± 78.82	117.60 ± 69.83	47.42 ± 71.29
Chinese standard		1000	350	200

Results are presented in dry weight as means ± SDs (n = 3). Means within a column with different superscript have differ significantly (*p* < 0.05).

*ND* not detectable.

Hg and Pb were not detected in all samples.

### Total trace element concentrations

The average concentration of total Cu in rice grains was 1.03 ± 0.53 mg kg^-1^. The FAO permissible limit for Cu in food is 40 mg kg^−1^. Therefore, the Cu concentration in all rice grain samples was lower than the permissible limit. The highest Cu concentration was red glutinous rice (2.50 mg kg^−1^) from Honghe, followed by soft rice 88 (1.51 mg kg^−1^) and purple rice (1.26 mg kg^−1^) from Dehong, while Cu content was undetectable in red and white rice from Lvchun, purple rice from Banna, Yunda 107 and Chujing 40 from Chuxiong, and white rice from Kunming (Table S1). The recommended nutrient intake (RNI) and tolerable upper intake level (UL) of Cu in foods are 0.8 mg d^-1^ and 8.0 mg d^-1^ for Chinese adults, respectively.

The average Fe concentration in rice was 4.80 ± 3.34 mg kg^-1^ (Table 1), which is lower than the allowable limit in rice (15 mg kg^-1^) and in food (5 mg kg^−1^, WHO /FAO 2011). The highest Fe content was found in Bianhua red soft rice from Dehong (11.49 ± 0.58 mg kg^−1^), while both Yunda 107 (0.56 mg kg^−1^) and Yunjing 37 (1.07 mg kg^−1^) from Chuxiong had lower Fe concentration. According to the Chinese Dietary Reference Intakes (NHCPRC 2017 ), the RNI for Fe in food for adults male are 12 mg d^−1^ and 20 mg d^−1^ for mg kg^−1^ body weight, respectively. UL for Fe in food for adults is 42 mg d^−1^ for mg kg^−1^ body weight.

The average Mn concentration of rice was 14.31 ± 21.66 mg kg^−1^. Chujing 39(105.70 ± 5.31 mg kg^−1^) was the highest followed by Mojiang purple rice (20.47 ± 0.85 mg kg^−1^) from Pu'er. Moreover, the lowest was detected in Yunda 107 (4.45 mg kg^−1^) from Chuxiong, followed by Kunming white rice (4.76 mg kg^−1^) (Table 1).

The mean total Zn concentration in rice samples was 13.48 ± 3.26 mg kg^−1^, which was below the allowable limit for cereals of United States Department Of Agriculture (50 μg g^−1^, USDA 2003) and in conventional foods (60 μg g^−1^, WHO /FAO 2001). The highest value of 18.82 ± 1.01 mg kg^−1^ was found in red glutinous rice1 from Honghe, while the lowest Zn content was found in rice Yunda 107 (7.64 ± 0.36 mg kg^−1^) from Chuxiong (Table 1). The RNI and UL for dietary Zn for adults in China are 12.5 mg d^−1^ and 40 mg d^−1^, respectively.

The average Se content in rice was measured to be 29.27 ± 26.14 μg kg^−1^. The highest content was found in Mojiang purple rice, followed by Bianhua red soft rice and Zhefang rice from Dehong, while Yunda 107 from Chuxiong had the lowest Se content (Table 1). Chinese standard GB/T 22499–2008 specifies that rice whose selenium content is less than 40 μg kg^−1^ is considered non-selenium-enriched rice; if it is above 0.3 mg kg^−1^, the selenium content exceeds the standard. Therefore, Mojiang purple rice, Bianhua red soft rice and Zhefang rice can be designated as selenium-rich rice varieties. The RNI and UL values for dietary selenium for adults in China were 40 μg d^−1^ and 400 μg d^−1^, respectively.

### Total macro element concentrations

The macro element analytical data of 20 rice varieties from different areas as shown in Supplementary Table 1. The average level of Ca in all the examined rice was measured at 69.62 ± 26.19 mg/kg. The greatest CA content was detected in Bianhua red soft rice (114.80 ± 1.97 mg kg^−1^) from Dehong, whereas Menghai fragrant rice from Xishuangbanna samples had the lowest Ca level (38.45 ± 0.79 mg kg^−1^). The adult estimated average requirement (EAR) of dietary Ca for Chinese residents is 650 mg d^-1^ for adults over 18 years old, and 800 mg d^-1^ for adults over 50 years old.

The average level of K in rice was measured at 1080.46 ± 603.31 mg kg^−1^. Bianhua red soft rice (2564.00 ± 65.05mg kg^−1^) and soft rice 88 (410.80 ± 13.75 mg kg^−1^) from Dehong have the greatest and lowest K level, respectively (Table 1). The adult adequate intake (AI) of dietary K for Chinese was 2000 mg d^−1^.

The average level of Mg in rice was measured at 491.60 ± 21.66 mg/kg. The greatest Mg content was detected in Bianhua red soft rice (1239.42 ± 20.95mg kg^−1^) from Dehong, whereasYunda 107 from Chuxiong had the lowest Mg level (110.39 ± 6.20 mg kg^−1^) (Table 1). The adult EAR and RNI of dietary Mg for Chinese was 280 mg d^−1^ and 330 mg d^−1^.

The average level of Na in rice was at 9.68 ± 5.73 mg kg^−1^. The greatest content was in Menghai fragrant rice (31.97 ± 2.72 mg kg^−1^) from Xishuangbanna, and the lowest level (6.06 ± 0.10 mg kg^−1^) was found in Banna purple rice also from Xishuangbanna (Table 1). The adult adequate intake (AI) of dietary Na for Chinese was 1500 mg d^−1^.

The average level of P in all rice samples was at 1635.29 ± 915.56 mg kg^−1^. The greatest P content was detected in Bianhua red soft rice (3341.38 ± 67.78mg/kg) from Dehong, whereas Yunda 107 from Chuxiong samples had the lowest level (697.45 ± 14.87 mg/kg) (Table 1). The adult RNI and UL of dietary P for Chinese residents is 720 mg d^−1^ and 3500 mg d^−1^.

### Toxic metal concentrations

Table 2 shows the results of the toxic element analysis. The average concentrations of Cr, As and Cd in rice were 236.83, 117.60 and 47.42 μg kg^−^1, respectively. Compared to the Chinese standard (GB 2762-2022) limit indicators for Cr, As and Cd concentrations, the average concentrations of the toxic metal were lower than the value of the Chinese standard value. However, Cd concentrations in Dehong purple rice and purple glutinous rice were above the Chinese standard limit.

### Origins differences of elements in rice

**Figure 1:**
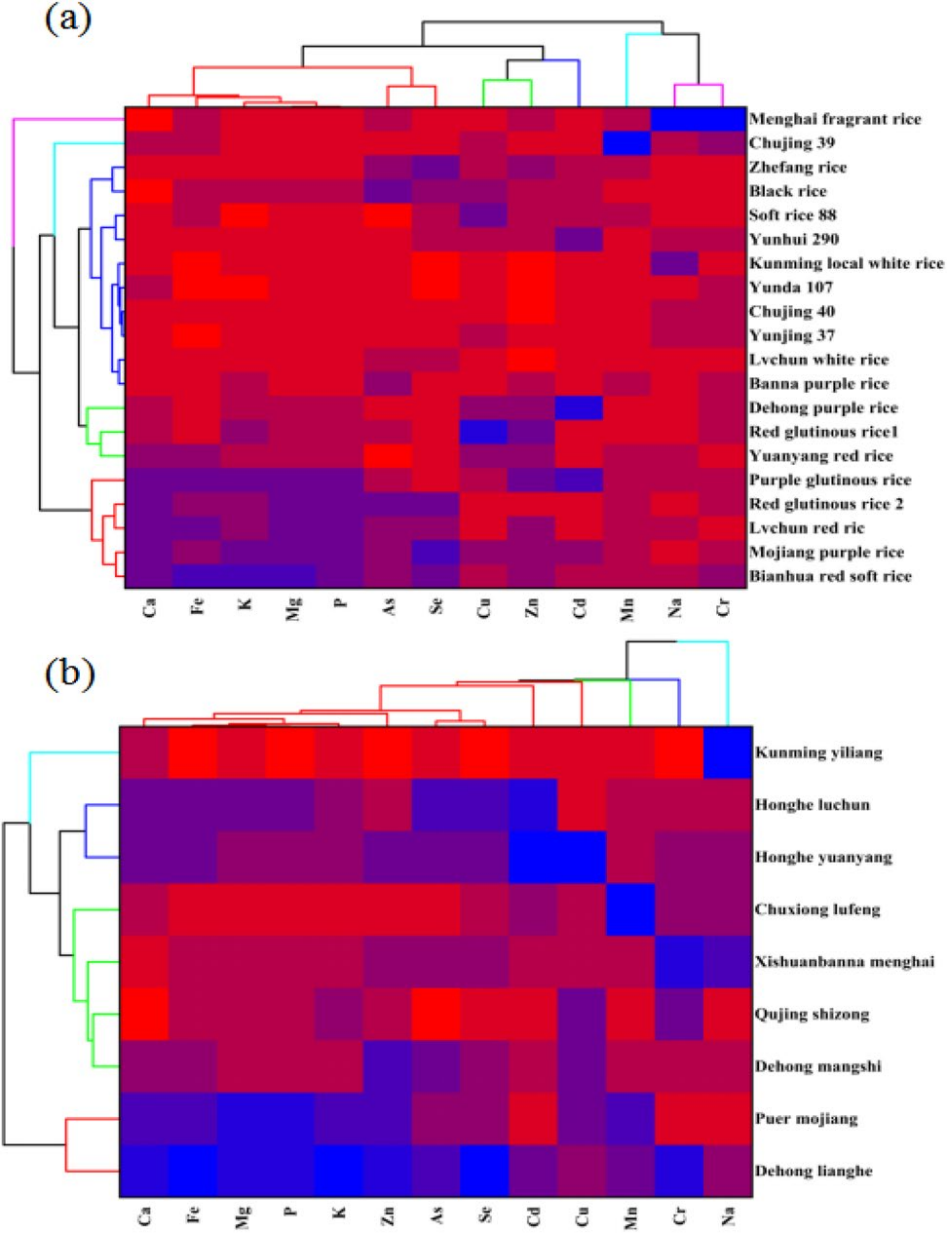
Heat map for rice samples. The blue to red colour indicates the high to low concentration. Figure 2a Heat map for rice varieties, Fig. 2b Heat map for rice geographical origins. Generated by Origin 2021 (Originlab, Massachusetts, USA).

The 20 rice varieties were classified into five categories based on the content of elements (Fig. 1a). Menghai fragrant rice was a separate category characterised by low Ca, high Na and Cr content, and Chujing 39 was also a separate category characterised by high Mn content. The three coloured rice varieties Dehong purple rice, red glutinous rice1 and Yuanyang red rice were grouped into a category characterised by high Cu, Zn and Cd content. Purple glutinous rice, red glutinous rice2, Ivchun red rice, Mojiang purple rice and Banna red soft rice were categorised as having high levels of Ca, Fe, K, Mg, P and Se. Other varieties were categorised as lower in Ca, Fe, K, Mg and high in As, Se, Cu, Zn and Cd.

**Figure 2:**
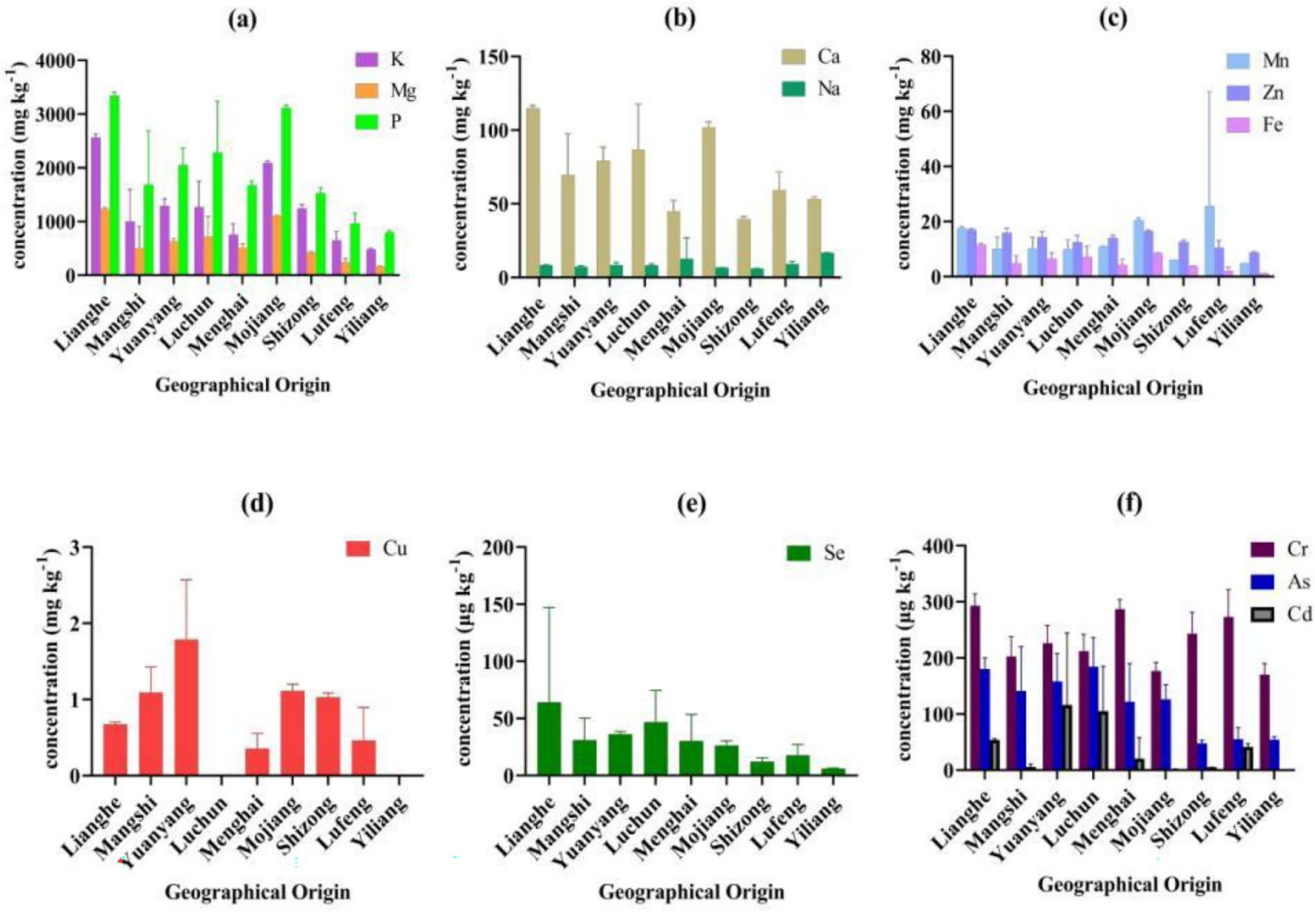
Element content of rice from different geographical origins.

There were large differences in the elements of rice from different origins, and can be divided into 4 categories (Fig. 1b). The lowest K (485.96 ± 11.13 mg kg^-1^), Mg (165.76 ± 10.26 mg kg^−1^), P (796.01 ± 34.06 mg kg^−1^), Mn (4.76 ± 0.09 mg kg^−1^), Zn (8.75 ± 0.25 mg kg^−1^), Fe (1.19 ± 0.05 mg kg^−1^), Se (6.10 ± 0.36 μg kg^−1^), Cr (170.00 ± 20.00 mg kg^−1^), Cd (ND) and the highest Na (16.74 ± 0.17 mg kg^−1^) were found in Kunming Yiliang white rice. This area should focus on the accumulation of micronutrients in rice between cultivation and processing. The second category was Lvchun and Yuanyang, where rice has high Cd content, as priority areas for heavy metal remediation. Honghe Yuanyang rice production had the highest Cu content (1.79 ± 0.78 mg kg^-1^), but Honghe Luchun rice samples were undetectable. The third category were Lufeng, Menghai, Shizong and Mangshi, lower levels of Ca, Fe, Mg, P, Zn, As and Se, also need to increase essential micronutrients. The highest Mn concentration was in Chuxiong Lufeng fong, while black rice from Qujing Shizong had the lowest levels of Ca (39.77 ± 1.54 mg kg^−1^), Na (6.09 ± 0.15 mg kg^-1^) and As (47.67 ± 5.51 μg kg^−1^). The fourth category was Mojiang and Lianghe with high contents of Ca, Fe, Mg, P, K, Zn and Mn. For example, rice from Dehong Lianghe had the highest contents of K (2564.00 ± 65.09 mg kg^−1^), Mg (1239.42 ± 20.95 mg kg^−1^), P (3341.38 ± 67.78 mg kg^−1^), Zn (17.00 ± 0.38 mg kg^−1^), Fe (11.49 ± 0.58 mg kg^−1^), Se (64.00 ± 83.14 μg kg^−1^) and Cr (293.33 ± 20.82 mg kg^−1^) (Fig. 2).

### Pigmented and non-pigmented rice

**Figure 3:**
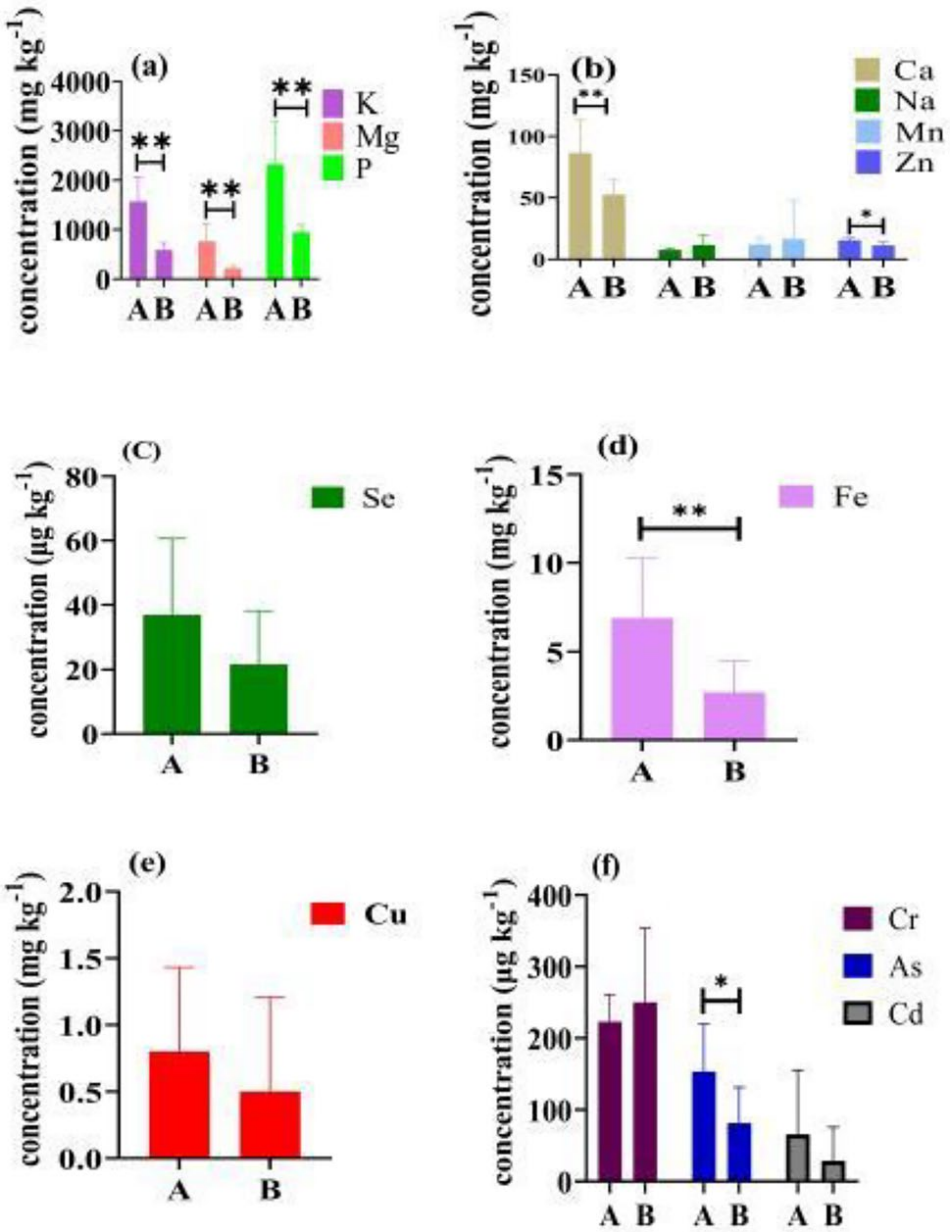
Comparison of element content between pigmented and non-pigmented rice. ***Indicates have significant difference at the 0.05 and 0.01 level, respectively; (**A**), (**B**) stand for pigmented rice and non-pigmented rice, respectively.

The results of the analysis showed that the element contents were quite different between pigmented and non-pigmented rice. Compared with pigmented rice, Na, Mn and Cr were higher in non-pigmented rice, the differences were not significant. In addition, with significant differences in P, K, Mg, Ca, Fe (P < 0.01), Zn and As (P < 0.05) (Fig. 3). In general, pigmented rice has a higher content of minerals, but this may cause the content of toxic elements to exceed the limit. In the samples of Red Glutinous Rice 2 and Black Rice, the As content is up to 240 μg kg^−1^ , which is above the limit of the international standards. The Cd content in Purple Glutinous Rice and Dehong Purple Rice (206.67 μg kg-1 and 250 μg kg^-1^) exceeds the limit of the Chinese standard (Table 2).

### Estimation of dietary intakes of mocro and trace elements from rice

The daily intakes of minerals in rice were estimated and compared with the RNI and UL values of the Chinese reference intakes for adults aged 18–50 years (Table S1). Daily intakes of minerals were calculated as mean body weight multiplied by daily rice consumption (0.346 kg). The Cu intake of sample No. 3 (red glutinous rice1) exceeded the RNI limit. The Mg intake from samples 1, 4, 5, 8 and 10 exceeded the RNI limit. With the exception of samples No. 13 and No. 15, the Mn intake from the other samples exceeded the RNI limit. The P uptake from samples 1, 4, 5, 8 and 10 all exceeded the RNI limit. All rice varieties exceeding the RNI limit were pigmented rice. For the minerals Ca, Cu, Fe, Na and Zn, there is a risk of inadequate intake, especially for non-pigmented rice, where almost all elements were inadequately absorbed. Compared with the RNI and UL (Table S1), the daily intake of Cr for adults exceeds the safe limit according to the Chinese reference intake. It indicates that the long-term large consumption of rice will result in the high exposure of Cr in Yunnan.

### Health risk of heavy metals

Health risk indicators were calculated for each rice variety using the equations. Supplementary Table S2 shows the values of the indicators estimated daily intake amount (EDI), hazard Quotient (HQ) and Hazard index (HI) for the population. The EDI of heavy metals was higher in females than in males. The EDI of Cr was the highest, and Menghai fragrant rice had the highest value in males (2.59 × 10^−3^ mg kg^−1^ d^−1^) and females (3.05 × 1^−3^ mg kg^−1^ d^−1^), respectively. Among the rice varieties, the samples of red glutinous rice 2 and Black rice had the highest EDI value of As, while the samples of Yuanyang red rice had the lowest EDI value. For all rice samples, the EDI value for Cd was highest in the samples of Dehong purple rice, while Yuanyang red rice and Kunming white rice had the lowest EDI value. The EDI value of Cd for a single rice ranged from 0 to 12.43 × 10^−4^ mgkg^−1^ d^−1^. Considering rice color, nonpigmented rice was found to have the higher average EDI value of Cr (1.24 × 10^−3^ mg kg^−1^ d^−1^ in males and 1.47 × 10^−3^ mg kg^−1^ d^−1^ in females, respectively), whereas pigmented rice had 1.11 × 10^−3^ mg kg^−1^ d^−1^ in males and 1.31 × 10^−3^ mg kg^−1^ d^−1^ in females. In contrast to pigmented rice with the highest average EDI of As and Cd, the EDI of As was 7.63 × 10 ^−4^ mg kg^−1^ d^−1^ in males and 9.01 × 10^−4^ mg kg^−1^ d^−1^ in females, respectively. For non-pigmented rice, the value was 4.06 × 10^−4^ mg kg^−1^ d^−1^ in males and 4.79 × 10^−4^ mg kg^−1^ d^−1^ in females, respectively. As Cd, the EDI of pigmented rice was calculated as 3.28 × 10^−4^ mg kg^−1^ d^−1^ in males and 3.86 × 10^−4^ mg kg^−1^ d^−1^ in females, respectively, and of non-pigmented rice as 1.44 × 10^−4^ mg kg^−1^ d^−1^ in males and 1.70 × 10^−4^ mg kg^−1^ d^−1^ in females, respectively.

The THQ and HI assessed are summarized in Supplementary Table S2. The THQ for Cr ranged from 1.27 × 10^−3^ in males and 1.50 × 10^−3^ in females for Yuanyang red rice and Kunming white rice, and 3.88 × 10^−3^ in males and 4.57 × 10^−3^ in females for Menghai fragrant rice. Red glutinous rice 2 and black rice were the samples with the highest THQ for As, in contrast to Yuanyang red rice, where the THQ was the lowest, while the highest THQ for Cd ranged from 0 in Yuanyang red rice and Kunming white rice to 7.13 × 10^−7^ in males and 8.41 × 10^−7^ in females in the samples from Yunhui 290. The mean total THQ for Cr of in nonpigmented rice (1.87 × 10^−3^ in males and 2.20 × 10^−3^ in females) was higher than in pigmented rice (1.67 × 10^−3^ in males and 1.96 × 10^−3^ in females, respectively). In contrast to Cr, the mean THQs for As and Cd were higher in pigmented rice than in nonpigmented rice. The THQ of pigmented rice for As (2.29 × 10^−7^ in meals and 2.70 × 10^−7^ in femeals, respectively), whereas non-pigmented rice had the lower THQ (1.22 × 10^−7^ in meals and 1.44 × 10^−7^ in femeals, respectively). Analysis of the THQ for Cd in pigmented rice products, showed a THQ of 3.28 × 10^−7^ in meals and 3.86 × 10 ^-7^ in female meals, respectively, while the THQ for non-pigmented rice was 1.44 × 10^−7^ in male meals and 1.70 × 10^−7^ in female meals.

The HI value was used to indicate the total exposure due to the intake of the toxic elements in rice. In our result, it was estimated to be 1.27 × 10^−3^ to 3.88 × 10^−3^ in mals and from 1.50 × 10^−3^ to 4.57 × 10^−3^ in females, respectively, doesn't pose an increased health risk (the reference value was < 4). However, Menghai fragrant rice has the highest value of exposure to the toxic elements, while Yuanyang red rice and Kunming white rice have the lowest. Analyzed separately, HI proved to be lower in pigmented rice sample (1.67 × 10^−3^ in males and 1.97 × 10^−3^ in females, respectively) than non-pigmented rice sample (1.87 × 10^−3^ in males and 2.20 × 10^−3^ in females, respectively).

The CR and TCR for toxic elements with carcinogenic risk were estimated in Table 3. Our results show that the cancer risk from consumption of the studied rice products is all greater than 10^−4^. Overall, CR was was lower in males than in females, with Cr, As and Cd values of 5.9 × 10^−4^, 8.8 × 10^−4^, and 14.3 × 10^−4^ in males and 6.9 × 10^−4^, 10.3 × 10^−4^, and 17.0 × 10^−4^ in females, respectively. TheTCR value was 0.0029 and 0.0034 for males and females, respectively, indicating a high potential cancer risk from research rice. The toxic heavy metal elements CR value were as follows Cd > As > Cr.

**Table 3:** Carcinogenic risks estimated for studied rice.

Type of Rice Product	CR						TCR	
	Cr		As		Cd			
	Males	Females	Males	Females	Males	Females	Males	Females
Bianhua red soft rice	7.3 × 10^−4^	8.6 × 10^–4^	13.4 × 10^–4^	15.8 × 10^–4^	16.3 × 10^–4^	19.2 × 10^–4^	37.0 × 10^–4^	43.6 × 10^–4^
Yuanyang red rice	4.2 × 10^−4^	5.0 × 10^–4^	2.5 × 10^–4^	3.0 × 10^–4^	0	0	6.8 × 10–4	8.0 × ^10–4^
Red glutinous rice1	5.5 × 10^−4^	6.5 × ^10–4^	9.2 × 10^–4^	10.8 × 10^–4^	3.7 × 10^–4^	4.4 × 10^–4^	18.4 × 10^–4^	21.7 × 10^–4^
Lvchun red rice	4.5 × 10^−4^	5.3 × 10^–4^	12.4 × 10^–4^	14.7 × 10^–4^	1.0 × 10^–4^	1.2 × 10^–4^	17.9 × 10^–4^	21.1 × 10^–4^
Red glutinous rice 2	6.0 × 10^−4^	7.0 × 10^–4^	17.9 × 10^–4^	21.1 × 10^–4^	2.4 × 10^–4^	2.8 × 10^–4^	26.3 × 10^–4^	31.0 × 10^–4^
Banna purple rice	6.1 × 10^–4^	7.2 × 10^–4^	12.2 × 10^–4^	14.4 × 10^–4^	5.0 × 10^–4^	5.8 × 10^–4^	23.3 × 10^–4^	27.4 × 10^–4^
Dehong purple rice	6.1 × 10^−4^	7.2 × 10^–4^	5.6 × 10^–4^	6.6 × 10^–4^	75.8 × 10^–4^	89.4 × 10^–4^	87.5 × 10^–4^	103.3 × 10^–4^
Mojiang purple rice	5.7 × 10^−4^	6.7 × 10^–4^	13.9 × 10^–4^	16.4 × 10^–4^	24.1 × 10^–4^	28.4 × 10^–4^	43.7 × 10^–4^	51.5 × 10^–4^
black rice	4.6 × 10−^4^	5.4 × 10^–4^	17.9 × 10^–4^	21.1 × 10^–4^	8.9 × 10^–4^	10.5 × 10^–4^	31.3 × 10^–4^	37.0 × 10^–4^
Purple glutinous rice	5.6 × 10^−4^	6.5 × 10^–4^	9.4 × 10^–4^	11.1 × 10^–4^	62.7 × 10^–4^	73.9 × 10^–4^	77.7 × 10^–4^	91.6 × 10^–4^
Zhefang rice	5.1 × 10^−4^	6.1 × 10^–4^	14.7 × 10^–4^	17.3 × 10^–4^	21.7 × 10^–4^	25.6 × 10^–4^	41.5 × 10^–4^	49.0 × 10^–4^
Menghai fragrant rice	12.9 × 10^−4^	15.2 × 10^–4^	8.7 × 10^–4^	10.3 × 10^–4^	1.1 × 10^–4^	1.3 × 10^–4^	22.7 × 10^–4^	26.8 × 10^–4^
Lvchun white rice	4.4 × 10^−4^	5.2 × 10^–4^	9.4 × 10^–4^	11.1 × 10^–4^	5.9 × 10^–4^	0.69 × 10^–4^	14.4 × 10^–4^	17.0 × 10^–4^
Yunjing 37	6.0 × 10^−4^	7.1 × 10^–4^	3.6 × 10^–4^	4.2 × 10^–4^	1.5 × 10^–4^	1.8 × 10^–4^	11.1 × 10^–4^	13.1 × 10^–4^
Yunda 107	5.6 × 10^−4^	6.5 × 10^–4^	4.6 × 10^–4^	5.5 × 10^–4^	0.90 × 10^–4^	1.1 × 10^–4^	11.1 × 10^–4^	13.1 × 10^–4^
Yunhui 290	5.4 × 10^−4^	6.4 × 10^–4^	4.0 × 10^–4^	4.7 × 10^–4^	43.5 × 10^–4^	51.3 × 10^–4^	52.9 × 10^–4^	62.3 × 10^–4^
Chujing 39	8.0 × 10^−4^	9.5 × 10^–4^	4.7 × 10^–4^	5.6 × 10^–4^	3.6 × 10^–4^	4.3 × 10^–4^	16.4 × 10^–4^	19.4 × 10^–4^
Chujing 40	5.5 × 10^−4^	6.5 × 10^–4^	4.5 × 10^–4^	5.3 × 10^–4^	0.62 × 10^−4^	0.73 × 10^–4^	10.6 × 10^–4^	12.5 × 10^–4^
Soft rice 88	5.1 × 10^−4^	6.0 × 10^–4^	2.6 × 10^–4^	3.1 × 10^–4^	14.3 × ^10−4^	16.8 × ^10−4^	21.9 × 10^–4^	25.9 × 10^–4^
Kunming local white rice	4.2 × 10^−4^	5.0 × 10^−4^	4.1 × 10^–4^	4.8 × ^10−4^	0	0	8.3 × ^10-4^	9.8 × 10^–4^
Average	5.9 × 10^−4^	6.9 × 10^−4^	8.8 × 10^−4^	10.3 × 10^−4^	14.4 × 10^–4^	17.0 × 10^–4^	29.0 × 10^–4^	34.3 × 10^–4^

**Figure 4:**
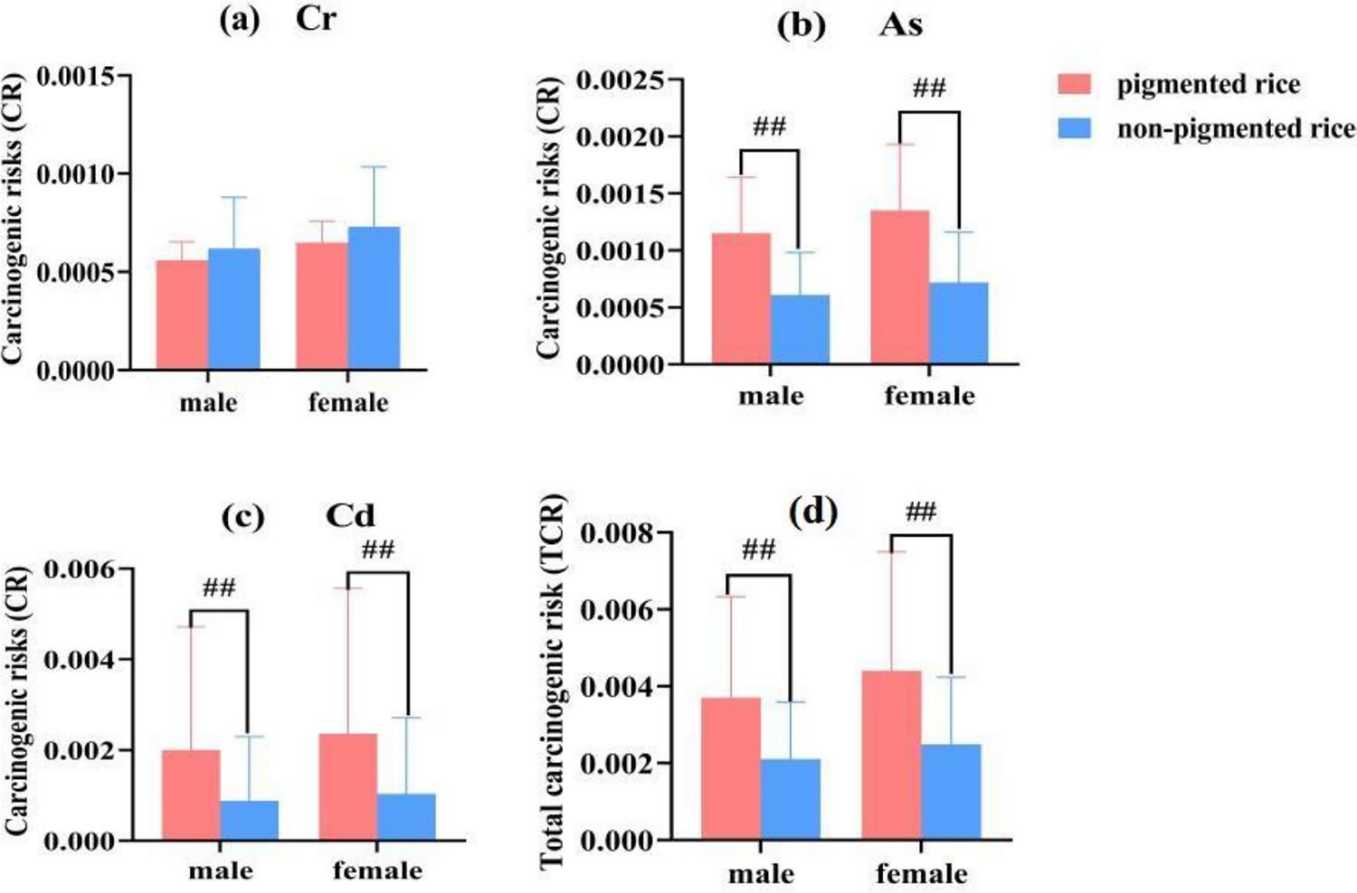
Carcinogenic risk comparison between pigmented and non-pigmented rice. ##Indicates have significant difference at the 0.01 level.

Carcinogenic risks between pigmented and non-pigmented rice. According to our results, According to our results, both male and female CR values for Cr were higher in non-pigmented rice than in pigmented rice, but no significant difference was observed (Fig. 4a). In contrast, CR values of As and Cd were higher in males and females in pigmented rice and differed significantly from those in non-pigmented rice (p < 0.01) (Fig. 4b,c). In non-pigmented rice, the CR values for As and Cr were 6.1 × 10^−4^, 8.8 × 10^−4^ in males and 7.2 × 10^−4^, 10.4 × 10^−4^ in females, respectively. For pigmented rice, the values were 11.5 × 10^−4^, 20.0 × 10^−4^ for males and 13.5 × 10^−4^, 23.6 × 10^−4^ for females. The TCR value also showed the same trend: pigmented rice was significantly higher than non-pigmented rice (Fig. 4d). In non-pigmented rice, the TCR value was 21.1 × 10^−4^ and 24.9 × 10^−4^ for males and females, respectively. In contrast, the values for pigmented rice were 37.0 × 10^−4^ for males and 43.6 × 10^−4^ for females.

## Discussion

### Elemental concentrations in rice

China's Yunnan province is one of the largest centers of rice seeds genetic and ecological diversity in the world^27^. Many rice varieties have high quality and special characteristics and are protected as a geographical name. At the same time, Yunnan Province have rich mineral resources in China, and the background value of soil elements in cultivated land has high value regionally. The background values of Pb, Zn, Cd, Hg, and As are high in most cultivation areas, and the background values of elements such as As, Cd and Cu are higher than the average in China ^28,29^.

There are different levels of elements accumulation between rice subspecies, an also have correlation patterns among a number of elements^30^. Enrichment index of heavy metals and elemental in rice were significantly correlated with soil properties. Soil pH and organic matter were the most important factors controlling the uptake of heavy metals by rice^13^.

Due to the status of rice as a staple food, people pay more attention to the essential minerals and the harmless levels of some toxic elements. Comparing the RNI for an adult Chinese, it can be seen that the average intake of Mn and P from pigmented rice meets the intake requirements but does not contain sufficient Ca, Cu, Fe, K, Mg, Na, Zn, and Se to meet the dietary requirements (Table S1). The deficiency of essential elements in rice appears to be a worldwide problem. Joy^31^ reported that rice from Malawi does not contain enough Ca, I, Se, or Zn to meet nutritional requirements. Tyagi^32^ indicated that rice from the Gangetic Basin in India may not provide people with the recommended daily requirement of Fe. Although rice is known to contain various minerals, the mineral content varies due to differences in geographical region associated with soil quality^33^, and appropriate dietary adjustment should be made to compensate for the lack of element intake.

The content of elements in rice was influenced by the degree of polishing. The outer layer of rice grains contains significant amounts of minerals such as K, Ca, Mg, and Fe^34^. Research by Jo^35^ showed that due to the different element distribution in the outer layer and endosperm of rice, elemental concentrations are higher in brown rice compared to 100% polished rice (white rice) for all elements except S and Se during the polishing process. Šlejkovec^36^also reported that the element concentrations of Mg, Mn, P, Fe, and K in brown rice were 2–4 times higher than in polished rice from the Slovenian market. In China, pigmented rice is mostly consumed in the form of brown rice, retaining most of the mineral elements, functional components and antioxidant activity, and increaseing the risk of toxic element concentrations exceeding the permissible limits. Nookabkaew^37^ have reported that non-polished rice had higher Mg, Ca, Mn, Fe and Se concentrations than polished rice in Thailand. In our study, the content of K, Mg, P, Ca, Zn, Fe and As elements in pigmented rice was significantly higher than that in non-pigmented rice (Fig. 3), our results were consistent with the above studies. However, the element content in rice is closely linked to the element content in the soil. The limitation of this study is that the soil properties of rice plants were not analysed. In future studies, soil properties will need to be tested in order to provide more help for risk assessment.

### Dietary intake and health risk assessment of elemental in rice

Macro, trace element and heavy metal contamination in rice grains is a growing concern. Rice is a poor source of minerals, and people on rice diets are at high risk of mineral deficiencies^38^. Research have recommends fortifying rice with iron, zinc, and other vitamins^39^.

Research has shown that health risks from PTEs in rice vary by age, gender, and milling grade. Children and women are more susceptible to heavy metal exposure^40^. Red rice, purple rice, and black rice are usually consumed in the form of paddy rice; other rice varieties have a higher degree of processing and are usually consumed in the form of polished rice, with the degree of milling making the difference in element content and loss of elements during processing^41,42^.

The Se concentration of Se-rich rice in China ranged from 0.012 ± 0.001 to 0.558 ± 0.057 mg/kg with an average of 0.090 ± 0.092 mg/kg^43^. In our study, the average Se concentration was 0.029 ± 0.026 mg/kg (Table 1), which was lower than the national average. Rice samples with Se concentrations below 0.04 mg/kg accounted for 30% of the total samples. Using the upper tolerable limit of 400 μg/d as a risk standard, the risk of selenium intake from selenium-rich rice was low (Table S1).

In this study, although heavy metal concentrations in some samples were above the limit, the results of THQ and HI (Table S2) were within the safe level (< 1 ) in all the samples, so comsumption of rice didn't pose a potential health risk. Therefore, there was no serious non-carcinogenic risk to human health from exposure to metals through consumption of this rice.

## Conclusion

The mean contents of P, K, Mg, Ca, Na, Mn, Zn, Fe, Se and the toxic heavy metals Cr, As, Cd, Hg and Pb in rice from Yunnan,China were analysed. Pigmented rice contents more macro minerals P, K, Mg and Ca, trace elements Zn, Fe, Cu and Se and toxic elements As, Cd compared to non-pigmented rice. A small part of the Cd and As content of pigmented rice exceeds the standard. The content of minerals Ca, Cu, Fe, Na and Zn in rice was far below the RNI, especially in non-pigmented rice. The health risk assessment of PTEs also showed that the carcinogenic risk is not acceptable. The CR value with PTEs was as follows Cd > As > Cr, and the CR and TCR values of As, Cd for non-pigmented rice were significantly lower than for pigmented rice. People may be at higher health risk from pigmented rice, especially women. Therefore, the long-term consumption of local rice should be reduced, and measures need to be taken to increase micronutrient levels and control levels of PTEs.

## Materials and methods

### Rice sample preparation

**Figure 5:**
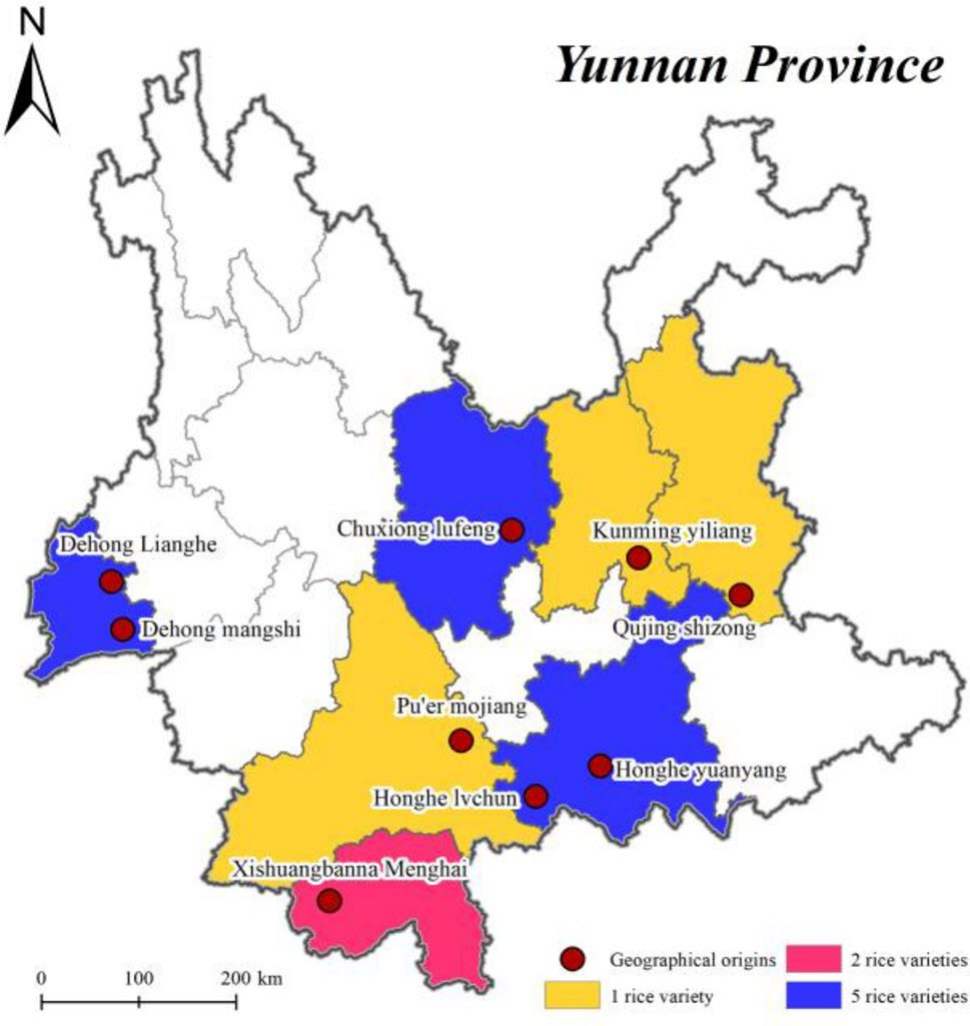
Geographical origin of rice samples. Generated by ArcGIS 10.2.

20 rice samples were collected from local rice production farms in Yunnan, harvested in 2020 and 2021, weighing 3 kg each. About 100 g of each type of original whole-grain rice was mixed by uniform grinding in a high-speed mill and passed through an 80-mesh sieve and dried at 80℃ in a hot-air oven to a constant weight and then stored in a desiccator at room temperature. Figure 5 shows the geographical origin of researched rice samples and photographs of the different rice samples can be found as Supplementary Figure S1 online.

### Samples analysis

Total concentrations of macro, trace, and toxic elements were analyzed by the closed wet digestion method. Approximately 1 g (accurate to 0.001 g) of the dry sample is weighed into polytetrafluoroethylene digestion vessel, then 10 ml of a mixed solution of nitric acid and perchloric acid (10:1) (ACS reagent, > 90%) is added, then heated from 25 °C to 95 °C for 45 min and held at 95 °C for 90 min using a hot plate until the digestion solution is colorless and transparent or slightly yellow, removed and cooled to room temperature, then diluted to 25 ml with water and shaken well. All samples were digested in triplicate during rice component metal analysis, and a blank test was performed simultaneously.

The digested solution was analysed for the content of Pb, Cd, Cr, As, Hg, Cu, Zn, Fe, Ca, K, Mg, Mn, Na, P, and Se by inductively coupled plasma mass spectrometry (ICP-MS) (PerkinElmer SCIEX ELAN DRC-e, Massachusetts, USA). The specific working parameters were set as follows: Automatic lens mode on, peak-hopping measurement mode, dwell time of 50 ms, 10 sweeps/measurement, 1500 W power, sample feed rate of 0.87 ml min^−1^. The concentrations of macro and trace minerals and toxic metals in the samples were expressed in mg kg^−1^ and μg kg^−1^ on a dry weight basis, respectively. The elemental content of the samples was quantified using the external standard method of multi-element standard solution (SGB-YYA230011). A rigorous quality control programme was also implemented to ensure the accuracy of the analytical results. This included blank samples, certified wheat flour reference material (GBW (E) 100,493) and the mean blank value was subtracted from the measured values before the result was calculated.

### Non-carcinogenic risk assessment

The estimated daily intake amount (EDI, mg/kg day) and Hazard Quotient (HQ) were used to determine the dose of oral exposure to harmful substances using the following Eqs. (1) and (2), respectively based on United States Environmental Protection Agency (2014). When THQ > 1, express certain toxic elements ingestion excess of the provisional maximum tolerable daily intake. The hazard index (HI) is the hazard index, used to assesse multiple heavy metals synergistic effect in rice, which was calculated as the sum of HQ. If *HI* < 1, chronic risks is acceptable, conversely, HI > 1, whereas non-cancer risks are likely to occur in the target population.

1 $$EDI = (C \times IR \times EF \times ED)/(BW \times AT)$$

2 $$THQ = EDI/RfD$$

3 $$HI = \sum\limits_{i = 1}^{n} {HQ_{i} }$$

where *EDI* (average dietary intake (mg kg^−1^ d^−1^), *C* (metal concentration, were obtained from the ICP-MS), *IR* (the rice ingestion rate, (adults: 0.346 kg d^−1^, obtained from China statistical Yearbook (2021)^24^. *ED* (exposure duration, 77 years), *EF* (exposure frequency, 365 days/year), *BW* (reference body mass, obtained from the Report on Nutrition and Chronic Disease Status of Chinese Residents (2020), the average weight of adult males and females is 69.6 kg and 59 kg, respectively), and *AT* (the averaging exposure time (*ED* × 365 days/year). *RfD* represents the oral reference dose (mg kg^−1^ d^−1^), determined by United States Environmental Protection Agency (USEPA) for Cd, As and Cr were 0.001, 0.0003 and 1.5 mg kg^−1^ d^−1^, respectively.

### Carcinogenic risk assessment

Carcinogenic risk (CR) indicates the probability of developing cancer. Total carcinogenic risk (TCR) represents the carcinogenic risk caused by multiple carcinogens, which is the sum of the carcinogenic risk of carcinogens. When the value of CR and TCR higher than 10^−4^ means an unacceptable carcinogenic risk, and risk-reducing measures must be taken, if the value between 10^−6^ ~ 10^−4^, risk is acceptable. The CR and TCR was calculated according to the following equation:

4 $$CR = EDI \times SF \times 10^{ - 3}$$

5 $$TCR = \sum\limits_{i}^{n} {CR_{i} }$$

where *SF* is the carcinogenicity slope factor, with of Cr, As, and Cd were 0.5, 1.5 and 6.1, respectively^22,23^.

### Evaluation of macro and trace minerals contribution to the AI and UL Values

Dietary intake levels for macro and trace minerals from rice were compared with the recommended nutrient intake (RNI ) and tolerable upper intake level (UL) as recommended by the Chinese dietary reference intakes^25,26^. RNI is as safe intake level of nutrients, it express nutrient intake levels that meet the vast majority of individuals in a given gender, age, and physical condition group. UL is the maximum daily intake of nutrients, below this amount is not considered detrimental.

### Statistical analysis

ArcGIS 10.2 (ESRI, California, USA) was used to generate geographical origin of rice samples. Data are presented as means ± standard deviation (SD) of dry weight of triplicate measurements and were statistically analysed by Duncan one-way analysis of variance (ANOVA). Significant differences (p < 0.05) in these means were analysed using SPSS statistical software version 20 (IBM, Armonk, NY, USA). Heat map analyses were performed to find grouping of rice samples by element content to detect differences in element enrichment of rice with Origin 2021 (Originlab, Massachusetts, USA).

### Ethical approval

The study complies with local and national guidelines and regulation.

### Supplementary Information

Supplementary Information.

## Data Availability

All data supporting the findings of this study are available within the paper.
