# Supplementary material for: Assessment of macro, trace and toxic element intake from rice: differences between cultivars, pigmented and non-pigmented rice
Source: Sci Rep. 2024 May 6;14:10398. doi: 10.1038/s41598-024-58411-1 (PMC11074271; doi:10.1038/s41598-024-58411-1)
Supplement: Supplementary file 1 — Supplementary Information. [file 41598_2024_58411_MOESM1_ESM.docx]

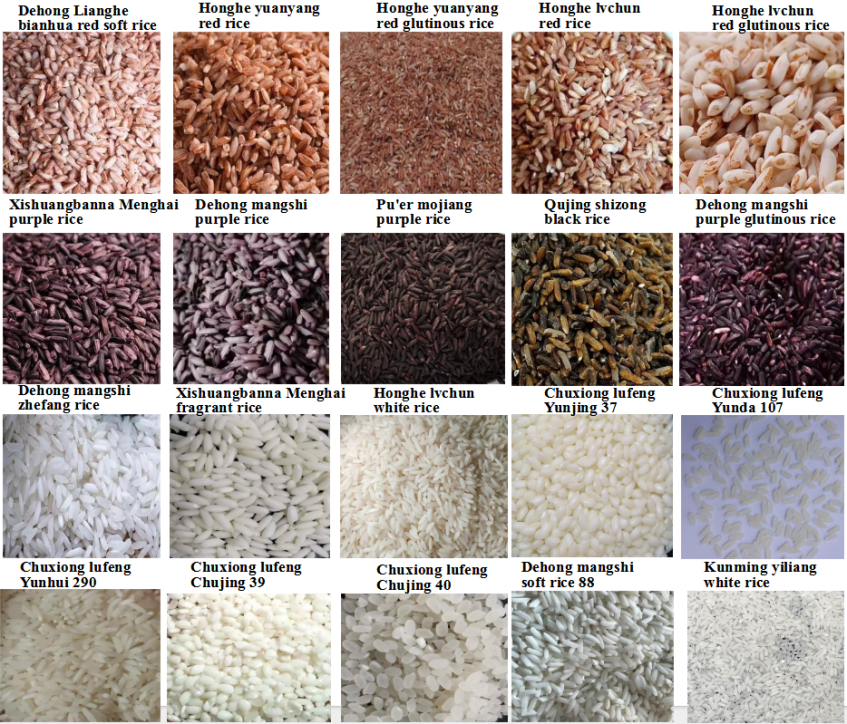


Figure S1 Photographs of the different rice samples

Table S1 Daily intake estimations for minerals in rice

| minerals | RNI (mg/Day) | | UL (mg/Day) | | Daily Intake of Sample(mg/Day) | | | | | | | | | | | | | | | | | | | |
| --- | --- | --- | --- | --- | --- | --- | --- | --- | --- | --- | --- | --- | --- | --- | --- | --- | --- | --- | --- | --- | --- | --- | --- | --- |
|  | female | male | female | male | 1 | 2 | 3 | 4 | 5 | 6 | 7 | 8 | 9 | 10 | 11 | 12 | 13 | 14 | 15 | 16 | 17 | 18 | 19 | 20 |
| Ca | 800 | 800 | 2000 | 2000 | 39.7 | 30.3 | 24.4 | 35.1 | 39.1 | 17.9 | 25.5 | 35.3 | 13.8 | 38.7 | 15.8 | 13.3 | 16.0 | 19.9 | 23.4 | 14.6 | 26.5 | 18.2 | 16.2 | 18.4 |
| Cu | 0.8 | 0.8 | 8.0 | 8.0 | 0.23 | 0.37 | 0.86 | 0 | 0 | 0 | 0.43 | 0.39 | 0.36 | 0.23 | 0.32 | 0.12 | 0 | 0.16 | 0 | 0.32 | 0.32 | 0 | 0.52 | 0 |
| Fe | 12 | 20 | 42 | 42 | 3.98 | 2.51 | 1.22 | 3.88 | 2.75 | 0.83 | 1.12 | 2.95 | 1.33 | 3.23 | 1.07 | 2.13 | 0.76 | 0.37 | 0.19 | 0.59 | 1.57 | 0.86 | 1.42 | 0.41 |
| K | 2000 | 2000 | Null | Null | 887 | 422 | 496 | 569 | 529 | 326 | 427 | 725 | 431 | 631 | 186 | 196 | 217 | 219 | 147 | 278 | 293 | 189 | 142 | 168 |
| Mg | 330 | 330 | Null | Null | 429 | 192 | 153 | 341 | 328 | 113 | 164 | 385 | 146 | 397 | 58 | 71 | 75 | 85 | 38 | 95 | 111 | 88 | 76 | 57 |
| Mn | 1.80^a^ | 2.30^a^ | 11^a^ | 11^a^ | 6.07 | 4.99 | 2.47 | 4.37 | 4.11 | 3.05 | 2.26 | 7.08 | 2.08 | 5.86 | 3.03 | 2.99 | 1.77 | 2.36 | 1.54 | 2.15 | 36.6 | 1.89 | 2.75 | 1.65 |
| Na | 1500 | 1500 | Null | Null | 2.92 | 3.62 | 2.61 | 3.29 | 2.68 | 2.10 | 2.10 | 2.30 | 2.11 | 2.81 | 2.48 | 11.1 | 2.50 | 3.97 | 2.48 | 2.79 | 2.87 | 3.82 | 2.68 | 5.79 |
| P | 720 | 720 | 3500 | 3500 | 1156 | 689 | 492 | 1050 | 969 | 371 | 596 | 1078 | 530 | 1118 | 299 | 340 | 358 | 298 | 241 | 425 | 362 | 345 | 326 | 275 |
| Zn | 12.5 | 7.5 | 40 | 40 | 5.88 | 5.40 | 6.51 | 5.27 | 4.29 | 4.37 | 5.87 | 5.73 | 4.37 | 6.10 | 5.42 | 4.89 | 3.33 | 3.49 | 2.64 | 4.92 | 4.21 | 2.99 | 4.61 | 3.03 |
| Se^＊^ | 60 | 60 | 400 | 400 | 22.1 | 4.7 | 4.6 | 14.4 | 19.2 | 6.8 | 7.3 | 28.1 | 14.0 | 6.5 | 21.6 | 7.0 | 9.1 | 4.3 | 1.5 | 8.0 | 7.0 | 4.2 | 10.2 | 2.1 |
| Cr^＊^ | 0.03 | 0.03 | Null | Null | 0.10 | 0.059 | 0.076 | 0.062 | 0.083 | 0.085 | 0.085 | 0.080 | 0.063 | 0.077 | 0.072 | 0.18 | 0.061 | 0.084 | 0.077 | 0.075 | 0.11 | 0.076 | 0.070 | 0.059 |
| As^＊^ | Null | Null | 13.9^b^ | 11.8^b^ | 0.06 | 0.01 | 0.04 | 0.06 | 0.083 | 0.056 | 0.026 | 0.065 | 0.083 | 0.044 | 0.068 | 0.040 | 0.044 | 0.017 | 0.022 | 0.019 | 0.022 | 0.021 | 0.012 | 0.019 |
| Cd^＊^ | Null | Null | 49^c^ | 58^c^ | 0.019 | 0 | 0.004 | 0.001 | 0.003 | 0.006 | 0.087 | 0.027 | 0.010 | 0.072 | 0.025 | 0.001 | 0.67×10^-3^ | 1.73×10^-3^ | 0.001 | 0.050 | 0.004 | 0.70×10^-3^ | 0.016 | 0 |

Note:1-20 represent Bianhua red soft rice, Yuanyang red rice, Red glutinous rice1, Lvchun red rice, Red glutinous rice 2, Banna purple rice, Dehong purple rice, Mojiang purple rice, Black rice, purple glutinous rice, Zhefang rice, Menghai fragrant rice, Lvchun white rice, Yunjing 37, Yunda 107, Yunhui 290, Chujing 39, Chujing 40, soft rice 88 and Kunming local white rice, respectively.

"Null" stands for no relevant standard value was found.

^＊^the unit is μg/Day.

^a^ date from Food and Nutrition Board, 2001

^b^ The provisional tolerable weekly intake (PTWI) of 21 mg/kg bw (equivalent to 3000 μg/kg bw/day) according to JECFA, UL=3×bw (femal and male is 59 and 69.6 kg, respectively ).

^c^ PTMI 0.025 mg/kg bw on a monthly basis according to JECFA (2013).

Supplementary Table S2 The values of EDI, THQ and HI estimated for studied rice.

|  | EDI | | | | | | THQ | | | | | | HI | |
| --- | --- | --- | --- | --- | --- | --- | --- | --- | --- | --- | --- | --- | --- | --- |
|  | Cr | | As | | Cd | | Cr | | As | | Cd | |  |  |
| Type of Rice Product | males | females | males | females | males | females | males | females | males | females | males | females | males | females |
| Bianhua red soft rice | 1.46×10^-3^ | 1.72×10^-3^ | 8.95×10^-4^ | 10.56×10^-4^ | 2.67×10^-4^ | 3.15×10^-4^ | 2.19×10^-3^ | 2.58×10^-3^ | 2.68×10^-7^ | 3.17×10^-7^ | 2.67×10^-7^ | 3.15×10^-7^ | 2.19×10^-3^ | 2.58×10^-3^ |
| Yuanyang red rice | 0.85×10^-3^ | 1.00×10^-3^ | 1.69×10^-4^ | 1.99×10^-4^ | 0 | 0 | 1.27×10^-3^ | 1.50×10^-3^ | 0.51×10^-7^ | 0.60×10^-7^ | 0 | 0 | 1.27×10^-3^ | 1.50×10^-3^ |
| red glutinous rice1 | 1.09×10^-3^ | 1.29×10^-3^ | 6.13×10^-4^ | 7.23×10^-4^ | 0.61×10^-4^ | 0.72×10^-4^ | 1.64×10^-3^ | 1.94×10^-3^ | 1.84×10^-7^ | 2.17×10^-7^ | 0.61×10^-7^ | 0.72×10^-7^ | 1.64×10^-3^ | 1.94×10^-3^ |
| Lvchun red rice | 0.89×10^-3^ | 1.06×10^-3^ | 8.29×10^-4^ | 9.77×10^-4^ | 0.17×10^-4^ | 0.20×10^-4^ | 1.34×10^-3^ | 1.58×10^-3^ | 2.49×10^-7^ | 2.93×10^-7^ | 0.17×10^-7^ | 0.20×10^-7^ | 1.34×10^-3^ | 1.58×10^-3^ |
| Red glutinous rice 2 | 1.19×10^-3^ | 1.41×10^-3^ | 11.93×10^-4^ | 14.07×10^-4^ | 0.40×10^-4^ | 0.47×10^-4^ | 1.79×10^-3^ | 2.11×10^-3^ | 3.58×10^-7^ | 4.22×10^-7^ | 0.40×10^-7^ | 0.47×10^-7^ | 1.79×10^-3^ | 2.11×10^-3^ |
| Banna purple rice | 1.23×10^-3^ | 1.45×10^-3^ | 8.12×10^-4^ | 9.58×10^-4^ | 0.81×10^-4^ | 0.96×10^-4^ | 1.84×10^-3^ | 2.17×10^-3^ | 2.44×10^-7^ | 2.87×10^-7^ | 0.81×10^-7^ | 0.96×10^-7^ | 1.84×10^-3^ | 2.17×10^-3^ |
| Dehong purple rice | 1.23×10^-3^ | 1.45×10^-3^ | 3.73×10^-4^ | 4.40×10^-4^ | 12.43×10^-4^ | 14.66×10^-4^ | 1.84×10^-3^ | 2.17×10^-3^ | 1.12×10^-7^ | 1.32×10^-7^ | 0.12×10^-7^ | 0.15×10^-7^ | 1.84×10^-3^ | 2.17×10^-3^ |
| Mojiang purple rice | 1.14×10^-3^ | 1.35×10^-3^ | 9.28×10^-4^ | 10.95×10^-4^ | 3.94×10^-4^ | 4.65×10^-4^ | 1.72×10^-3^ | 2.02×10^-3^ | 2.78×10^-7^ | 3.28×10^-7^ | 3.94×10^-7^ | 4.65×10^-7^ | 1.72×10^-3^ | 2.02×10^-3^ |
| black rice | 0.91×10^-3^ | 1.08×10^-3^ | 11.93×10^-4^ | 14.07×10^-4^ | 1.46×10^-4^ | 1.72×10^-4^ | 1.37×10^-3^ | 1.61×10^-3^ | 3.58×10^-7^ | 4.22×10^-7^ | 1.46×10^-7^ | 1.72×10^-7^ | 1.37×10^-3^ | 1.61×10^-3^ |
| purple glutinous rice | 1.11×10^-3^ | 1.31×10^-3^ | 6.30×10^-4^ | 7.43×10^-4^ | 10.27×10^-4^ | 12.12×10^-4^ | 1.67×10^-3^ | 1.96×10^-3^ | 1.89×10^-7^ | 2.23×10^-7^ | 0.10×10^-7^ | 0.12×10^-7^ | 1.67×10^-3^ | 1.97×10^-3^ |
| Zhefang rice | 1.03×10^-3^ | 1.21×10^-3^ | 9.78×10^-4^ | 11.53×10^-4^ | 3.56×10^-4^ | 4.20×10^-4^ | 1.54×10^-3^ | 1.82×10^-3^ | 2.93×10^-7^ | 3.46×10^-7^ | 3.56×10^-7^ | 4.20×10^-7^ | 1.54×10^-3^ | 1.82×10^-3^ |
| Menghai fragrant rice | 2.59×10^-3^ | 3.05×10^-3^ | 5.80×10^-4^ | 6.84×10^-4^ | 0.17×10^-4^ | 0.21×10^-4^ | 3.88×10^-3^ | 4.57×10^-3^ | 1.74×10^-7^ | 2.05×10^-7^ | 0.17×10^-7^ | 0.21×10^-7^ | 3.88×10^-3^ | 4.57×10^-3^ |
| Lvchun white rice | 0.88×10^-3^ | 1.04×10^-3^ | 6.26×10^-4^ | 7.39×10^-4^ | 0.096×10^-4^ | 0.11×10^-4^ | 1.32×10^-3^ | 1.55×10^-3^ | 1.88×10^-7^ | 2.22×10^-7^ | 0.09.6×10^-7^ | 0.11×10^-7^ | 1.32×10^-3^ | 1.55×10^-3^ |
| Yunjing 37 | 1.21×10^-3^ | 1.43×10^-3^ | 2.37×10^-4^ | 2.80×10^-4^ | 0.25×10^-4^ | 0.29×10^-4^ | 1.81×10^-3^ | 2.14×10^-3^ | 0.71×10^-7^ | 0.84×10^-7^ | 0.25×10^-7^ | 0.29×10^-7^ | 1.81×10^-3^ | 2.14×10^-3^ |
| Yunda 107 | 1.11×10^-3^ | 1.31×10^-3^ | 3.08×10^-4^ | 3.64×10^-4^ | 0.15×10^-4^ | 0.17×10^-4^ | 1.67×10^-3^ | 1.96×10^-3^ | 0.93×10^-7^ | 1.09×10^-7^ | 0.15×10^-7^ | 0.17×10^-7^ | 1.67×10^-3^ | 1.96×10^-3^ |
| Yunhui 290 | 1.08×10^-3^ | 1.27×10^-3^ | 2.67×10^-4^ | 3.15×10^-4^ | 7.13×10^-4^ | 8.41×10^-4^ | 1.62×10^-3^ | 1.91×10^-3^ | 0.80×10^-7^ | 0.94×10^-7^ | 7.13×10^-7^ | 8.41×10^-7^ | 1.62×10^-3^ | 1.91×10^-3^ |
| Chujing 39 | 1.61×10^-3^ | 1.90×10^-3^ | 3.17×10^-4^ | 3.73×10^-4^ | 0.60×10^-4^ | 0.70×10^-4^ | 2.41×10^-3^ | 2.84×10^-3^ | 0.95×10^-7^ | 1.12×10^-7^ | 0.60×10^-7^ | 0.70×10^-7^ | 2.41×10^-3^ | 2.84×10^-3^ |
| Chujing 40 | 1.09×10^-3^ | 1.29×10^-3^ | 3.02×10^-4^ | 3.56×10^-4^ | 0.10×10^-4^ | 0.12×10^-4^ | 1.64×10^-3^ | 1.94×10^-3^ | 0.91×10^-7^ | 1.07×10^-7^ | 0.10×10^-7^ | 0.12×10^-7^ | 1.64×10^-3^ | 1.94×10^-3^ |
| soft rice 88 | 1.01×10^-3^ | 1.19×10^-3^ | 1.74×10^-4^ | 2.05×10^-4^ | 2.34×10^-4^ | 2.76×10^-4^ | 1.52×10^-3^ | 1.79×10^-3^ | 0.52×10^-7^ | 0.62×10^-7^ | 2.34×10^-7^ | 2.76×10^-7^ | 1.52×10^-3^ | 1.79×10^-3^ |
| Kunming local  white rice | 0.85×10^-3^ | 1.00×10^-3^ | 2.70×10^-4^ | 3.19×10^-4^ | 0 | 0 | 1.27×10^-3^ | 1.50×10^-3^ | 0.81×10^-7^ | 0.96×10^-7^ | 0 | 0 | 1.27×10^-3^ | 1.50×10^-3^ |
